# Gene Therapy Leaves a Vicious Cycle

**DOI:** 10.3389/fonc.2019.00297

**Published:** 2019-04-24

**Authors:** Reena Goswami, Gayatri Subramanian, Liliya Silayeva, Isabelle Newkirk, Deborah Doctor, Karan Chawla, Saurabh Chattopadhyay, Dhyan Chandra, Nageswararao Chilukuri, Venkaiah Betapudi

**Affiliations:** ^1^Neuroscience Branch, Research Division, United States Army Medical Research Institute of Chemical Defense, Aberdeen, MD, United States; ^2^Department of Medical Microbiology and Immunology, University of Toledo College of Medicine and Life Sciences, Toledo, OH, United States; ^3^Roswell Park Comprehensive Cancer Center, Buffalo, NY, United States; ^4^Department of Physiology and Biophysics, Case Western Reserve University, Cleveland, OH, United States

**Keywords:** gene therapy, viral vectors, modern medicines, diseases and disorders, clinical trials

## Abstract

The human genetic code encrypted in thousands of genes holds the secret for synthesis of proteins that drive all biological processes necessary for normal life and death. Though the genetic ciphering remains unchanged through generations, some genes get disrupted, deleted and or mutated, manifesting diseases, and or disorders. Current treatment options—chemotherapy, protein therapy, radiotherapy, and surgery available for no more than 500 diseases—neither cure nor prevent genetic errors but often cause many side effects. However, gene therapy, colloquially called “living drug,” provides a one-time treatment option by rewriting or fixing errors in the natural genetic ciphering. Since gene therapy is predominantly a viral vector-based medicine, it has met with a fair bit of skepticism from both the science fraternity and patients. Now, thanks to advancements in gene editing and recombinant viral vector development, the interest of clinicians and pharmaceutical industries has been rekindled. With the advent of more than 12 different gene therapy drugs for curing cancer, blindness, immune, and neuronal disorders, this emerging experimental medicine has yet again come in the limelight. The present review article delves into the popular viral vectors used in gene therapy, advances, challenges, and perspectives.

## Introduction

The human genome contains ~25,000 genes that encode a wide variety of proteins colloquially called the building blocks and workhorses of the cell to drive every biological process necessary for life and death (1–4). Though the genetic ciphering remains largely unchanged through generations, some genes go awry due to mutations, and disruptions or deletions (5). These underlying and inevitable genetic changes translate into altered protein functions affecting normal cell structures, functions, and their physiological roles manifesting into a serious disease or deficiency or disorder (6, 7). According to the Genetic and Rare Diseases Information Center (GARD) and Global Genes®, the leading rare disease patient advocacy organization in the world, dysfunctional genes account for 80% of the total 7,136 diseases reported to date. Nearly 30 million people in the United States alone and more than 300 million people in the rest of the world are affected by genetic diseases; unfortunately, half of them are estimated to be children. According to the National Center for Advancing Translational Sciences (NCATS), only 500 human diseases are treatable with an estimated 10,000 drugs available to date, underscoring the necessity to develop new drugs and treatment options.

Although a significant advancement has been made in developing modern medicine, including chemotherapy, radiation, and surgery, many drugs are synthetic chemicals designed to alter the body's chemistry and create dependency overtime, and offer only temporary relief by reducing disease symptoms and increasing lifespan. These issues are partly addressed by developing protein therapy based on transcription factors, signaling proteins, gene editing enzymes, growth factors, engineered protein scaffolds, hormones, blood factors, thrombolytes, antibodies, and antigens. Some of them, especially the monoclonal antibody-based drugs including Humira, Rituxan, Avastin, Herceptin, Remicade, Lucentis, Enbrel, Synazis, and several others, are being used to treat cancer, diabetes, autoimmune disorders, infectious diseases, and others (8). In fact, both protein and peptide-based drugs have emerged as a major class of therapeutics with nearly 380 marketed pharmaceuticals available in the world (9). However, these protein-based therapies are facing many challenges including low solubility and bioavailability, *in vivo* physicochemical instability, short circulating half-life, penetrability *in vivo*, biodistribution, and causing toxicity in large amounts (10–15). Another adverse effect of introducing therapeutic proteins into a patient's body is that it may result in severe immune responses, inflammation, and fever (16). To add to the woes, the production and manufacturing of high quality therapeutic proteins have become highly complex activity (17). In fact, more than 5,000 critical steps are involved in developing a single therapeutic protein (8). Therefore, the quotient of unpredictability is very high in developing both chemical and protein-based therapies. Gene therapy, on the other hand, leads to long-lasting production of the desired therapeutic protein and can localize protein expression to an area of the body, fixing the problem at its source (18). Also, prognosis for a large number of incurable diseases appears grim, which is why gene therapy presents itself as a breakthrough alternative with immense potential to provide a one-time treatment option for a complete cure as well as disease and disorder prevention. Gene therapy is an emerging experimental treatment that delivers functional genes into a patient's body to counter or replace malfunctioning ones, thus curing disease without pharmacological intervention, radiotherapy, or surgery. This modern approach has the potential to offer complete protection against lethal nerve gases (13, 19–22) and treat monogenic and cardiovascular diseases, immunodeficiency, cancer, and more (23–27). Apart from genetic defects, several other diseases that cannot be treated with drugs or antibodies can be cured with gene therapy. In addition, every prescribed and non-prescribed drug comes with unwanted side effects, ranging from minor discomfort to death. According to Drugwatch®, a non-profit drug information network and organization, an estimated four million patients in the USA alone visit doctors annually due to adverse effects of prescription drugs. Hence, gene therapy that aligns with the natural human genetic transcriptome has the potential to become an unquestionable choice for complete treatment of diseases, disorders, and infectious diseases.

Gene therapy appears simple in principle but involves identification of affected gene(s), cloning and loading of a wild type or recombinant healthy version in a suitable vector for optimal delivery and expression in the target cells or tissue and thus has seen its fair share of hurdles. Because it often uses repurposed viruses to deliver therapeutic genes, gene therapy has been caught in a vicious cycle for nearly two decades owing to immune response, insertional mutagenesis, viral tropism, off-target activity, unwanted clinical outcomes (ranging from illness to death of participants in clinical trials), and patchy regulations (23, 28–31). This led to a sharp decline in research funding for basic, preclinical development and vector production via individual investigators grants such as R01 and program grants. Thus, with limited information of preclinical data and vector production, the number of clinical trials conducted worldwide did not rise steadily from 1999 to 2015 (32). Furthermore, funding of the actual clinical trial was not guaranteed even vectors have been produced and certified for human use at significant cost. The American Society of Gene Therapy has taken lead in fixing this fragmented funding method by making many recommendations including the elimination of redundant regulatory processes and establishment of the National Gene Vector Laboratories (NGVL) to review vector production and toxicology. Now, with new technological advances in gene delivery and editing methods, increased enthusiasm of clinicians and drug companies, the advent of several viral-based drugs in the market, and the potential to provide a one-time treatment option without corrupting the genetic code, gene therapy is breaking free of this cycle. Undoubtedly, the resurgent interest in offering gene therapy-based treatments is one of the most defining developments in the pharmaceutical industry and is expected to have far-reaching implications on curing dangerous diseases in the future. With an estimated US $11 billion market in the next 10 years, both clinical trials and pharmaceutical industry are anticipated to benefit immensely from gene therapy. Here, we describe popular viral vectors used in gene therapy and gene therapy drugs available in the market.

## Gene Therapy and Its Kinds

While the idea of gene therapy has been around for the past 80 years, Professor William Szybalski's demonstration in 1962 on correcting a genetic defect by delivering foreign DNA into mammalian cells is regarded as its birth (33). The Food and Drug Administration (FDA) defines gene therapy as products that “mediate their effects by transcription and/or translation of transferred genetic material and/or by integrating into the host genome and that are administered as nucleic acids, viruses, or genetically engineered microorganisms,” and the European Medicines Agency (EMA) describes gene therapy medicinal product (GTMP) as a “biological medicinal product that contains an active substance which contains or consists of a recombinant nucleic acid used in or administered to humans to regulate, repair, replace, add or delete genetic sequences and its therapeutic, prophylactic or diagnostic effect relates directly to the recombinant nucleic acid sequence it contains, or to the product of genetic expression of this sequence” (32, 34). Typically, DNA, mRNA, siRNA, miRNA, and anti-sense oligonucleotides are the genetic materials used for therapeutic delivery into a defective target cell or tissue to restore a specific gene function or turn off a gene responsible for disease or disorder development (35). Other methods include swapping the mutated gene for a functional gene using homologous recombination, repairing the mutated gene using selective reverse mutation, and regulating the mutated gene (36). Gene therapy allows the delivery of therapeutic genetic material to any specific cell or tissue and or organs of the body for treatment.

Based on the type of cells or tissues targeted for gene delivery and treatment, gene therapy is divided into germ-line and somatic cell gene therapies. Germ-line gene therapy involves genetic manipulation of the reproductive cells sperm and egg to make heritable changes. The potential of germ line therapy was successfully demonstrated in mouse, rat, rabbit, sheep, cattle, goat, and pig (37–40) but not in humans because of a moratorium due to ethical reasons, lack of advanced tools, and societal consensus (41–46). However, with recent technological advances in genome editing and gene delivery methods, renewal of debates on revisiting germ line therapy appears not far from reality (47–50). Therefore, the present review is focused on somatic cell gene therapy.

## Somatic Cell Gene Therapy

**Figure 1:**
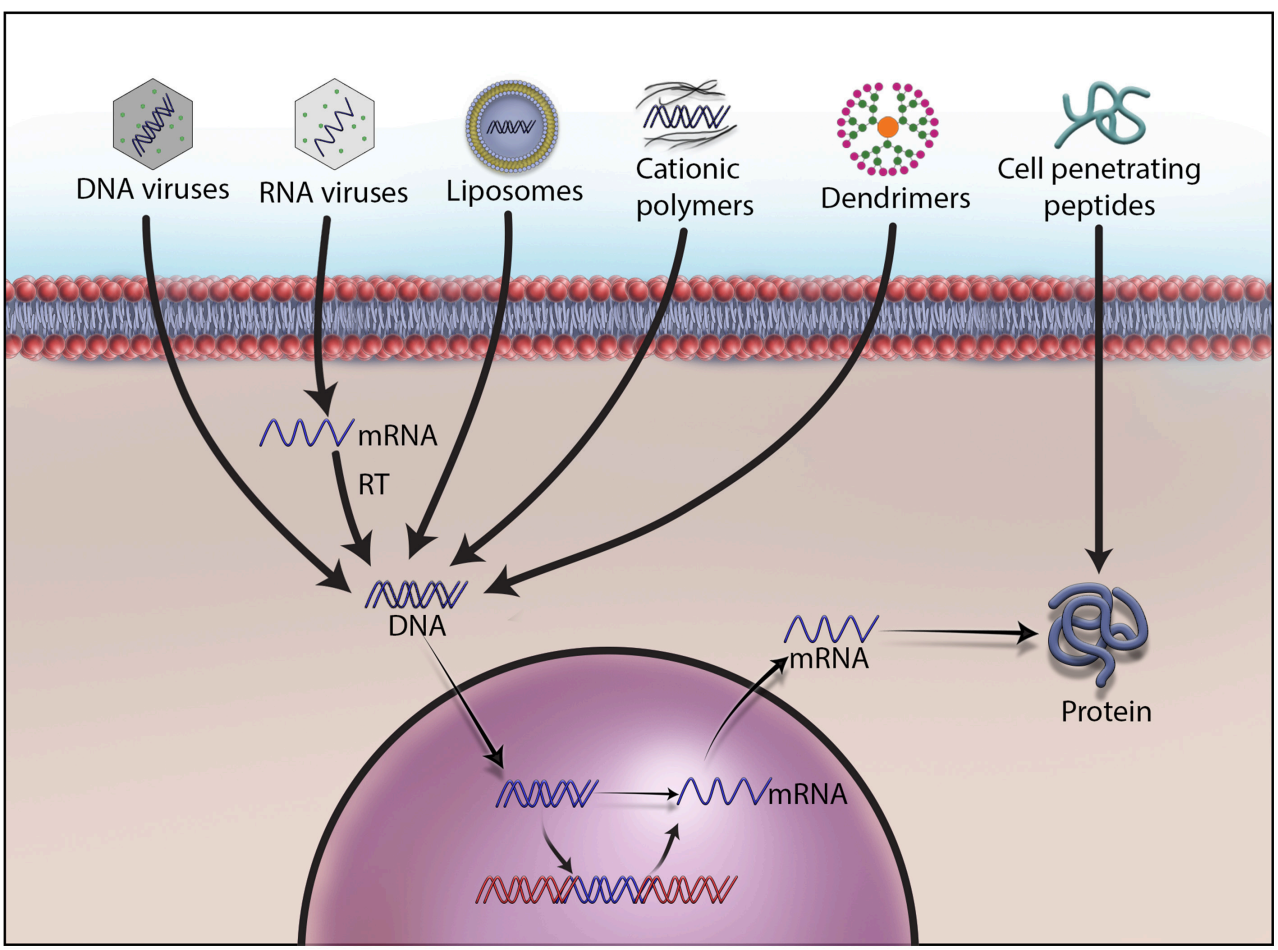
Different methods to deliver therapeutic DNA and proteins to target cells. Non-viral gene delivery methods have many advantages over viral vectors in gene therapy. They do not cause immunogenicity and carcinogenicity, and can deliver a large size of therapeutic DNA efficiently with a low price tag. As no one-size-fits-all solution to therapeutic DNA delivery exits, development, and formulations remain the main focus of research on non-viral methods.

In somatic cell gene therapy, every cell except sperm and egg is targeted for therapeutic treatment. It is considered safe because genetic changes remain in the patient and are not passed onto the offspring. However, the requirement of skill set and sophistication in delivering a therapeutic gene into the target cells or tissue of the patient elevates the quotient for an unpredictable clinical outcome. Therefore, many advanced methods are being developed to deliver therapeutic genetic materials, and they are broadly divided into *ex vivo, in situ*, and *in vivo* methods. *Ex vivo*, also called “outside the living body” method, involves isolating the cells to be treated from the patient, modifying them with a therapeutic gene, and re-introducing into the patient's body. Hepatocytes in the liver, retina photoreceptors in the eye, stem cells in the bone marrow, and T lymphocytes have been the focus of this method (43). Recently, the FDA has approved Kymriah™, a groundbreaking prescription cancer treatment that uses the patient's own white blood cells or T cells for inserting the CD19 gene *ex vivo* (51). After being re-introduced into the patient's blood, these genetically engineered T cells will have greater ability to target cancer cells. Less side effects than other methods, no risk of reaching germ-line cells, minimized immune response, and less renal clearance are other advantages of *ex vivo* method (52–54). Zalmoxis™ is another advanced somatic cell therapy product recently approved by the EMA for treating serious blood cancers such as certain types of leukemia and lymphomas. Zalmoxis™ consists of donor lymphocytes transfected with Herpes simplex virus-1 thymidine kinase (HSV-TK) and truncated low affinity nerve growth factor receptor (ΔLNGF). *In situ* delivery, or “in position” delivery, involves administration of the desired genetic material directly into the target cells or tissue. For example, Neovasculgen®, a plasmid vector carrying vascular endothelial growth factor (VEGF) gene, is directly injected into the target ischemic tissue to stimulate blood vessel growth (55–57). This method is being explored to cure cystic fibrosis, muscular dystrophy, and cancer but still requires more technological advancement in delivery methods for a successful clinical outcome (58–60). Though delivering genetic material by this method works well for localized conditions, it cannot be used for treating systemic disorders. The last and most important method of gene delivery is *in vivo*, or “in the living body.” In this method, viral, or non-viral vectors are used to deliver the therapeutic material to the defective target cells or tissue in the body (Figure 1). A wide variety of physical and chemical methods including needles, gene guns, electroporation, sonoporation, photoporation, magnetofection, hydroporation, mechanical massage, lipid, calcium phosphate, silica, and gold nanoparticles are being used to deliver genetic material to target cells. However, none of them is more efficient than viruses in delivering therapeutic genetic materials to the target cells due to their inherent shortcomings and operational complexity. The present review article is focused on viral vectors only.

## Viruses in Delivering Therapeutic Genes

There has been a quite bit of resentment in availing the benefits of viruses due to ignorance, bad rap, and skewed view. In fact, the human body offers shelter to viruses, fungi, protozoa, and worms by adopting appropriate mechanisms for mutual benefits in order to survive and thrive (61). For example, viruses offer immunity against bacterial pathogens and tumor cells, and modulate gut bacterial genes to improve host digestion (62). Though the word *virus* implies mortality and morbidity, viruses are considered nature's genetic engineers because of their ability to infect most kinds of organisms including bacteria, humans, animals, and plants. Also, viruses help certain plants to survive in extreme weather conditions (62). We have identified powerful viral promoters and enhancer elements that can be used to construct plasmid vectors for high level expression of foreign proteins (63, 64). They have an advantage over others by carrying several genes encoding structural and non-structural proteins to infect and propagate in host cells. Some viruses have the ability to transduce the cells they infect, i.e., stably express a gene along with the host's genome. They allow manipulation of their genome and removal of virulent genes without losing the ability to infect host cells. This makes them nearly dead or not alive, and the versatile biological entities, a pragmatic reason to accept them as sophisticated biological tools for delivering foreign genetic materials into eukaryotic cells. For example, we have manipulated and reconstituted Sendai viral envelopes containing only the fusion glycoprotein to deliver a reporter gene to liver *in vivo* (65). In fact, viral vectors were the first carriers of nucleic acids used in gene therapy (18).

Because of their abundance on the earth and difference in genetic makeup, many viruses are being used in preclinical and clinical investigations but each comes with its own unique advantages and disadvantages. Therefore, finding a suitable vector to deliver therapeutic genetic material has become a challenge to make gene therapy a viable and better treatment option than conventional methods. Part of the challenge is therapeutic DNA's inability to pass through the cell membrane because of its large size and negative charge. Also, the therapeutic DNA needs to escape the cellular endonucleases and renal clearance. An ideal vector should have enough space to transport large therapeutic genes, high transduction efficiency, and the ability to provide long-term and stable expression, as well as target specific cells, avoid random insertion of the therapeutic gene into the host genome, and infect mitotic as well as post-mitotic cells. It should not be immunogenic or pathogenic, should not cause inflammation and should possess the ability to be manufactured on a large scale. Research on developing novel viral vectors is advancing steadily with a special focus on substituting pathogenic genes with therapeutic DNA (66). In fact, non-pathogenic, replication-defective, and human-friendly viral vectors are being used in more than 70% of the ongoing gene therapy clinical trials worldwide (67). One particularly popular use of viral vectors, such as adenovirus, Seneca Valley virus, poliovirus, vaccinia virus, herpes simplex virus, reovirus, Coxsackievirus, parvovirus, Newcastle disease virus, vesicular stomatitis virus, and measles virus, is in the form of oncolytic viruses (OV). In 2016 alone, more than 40 clinical trials using OV were conducted (68). OV destroy malignant cancer cells by specifically replicating in those cells to effectively lyse them as well as induce a robust antitumor immune response. OV selectively replicate in tumor cells through a variety of methods such as virus-specific receptors on the cells. They can be used to deliver anti-angiogenesis genes, suicide genes, immunostimulatory genes, and DNA encoding small nucleic acids. Apart from carrying immunostimulatory genes, OV can induce an immune response by releasing cell debris and viral antigens (68). Many other innovative approaches are being developed to use viral vectors for treating diseases and disorders. Since Edward Tatum's initial proposal to repurpose viruses for therapeutic gene delivery in 1966, gene therapy has come a long way from the construction of many types of viral vectors to their use in more than 3,000 clinical trials to date (32, 69, 70). However, during this incredible journey with obscure regulations, gene therapy has experienced a few undesired clinical outcomes due to off-target effects, cytotoxicity, viral transmissibility, impurity, and an immune response to the viral vector itself (68). Nonetheless, diseases for which a cure has been attempted include β-thalassemia, X-linked severe combined immunodeficiency (X-SCID), adenosine deaminase deficiency (ADA-SCID), cystic fibrosis, hemophilia, liver enzyme ornithine transcarbamylase (OTC) deficiency, head and neck cancer, metastatic melanoma, HIV, Leber's congenital amaurosis, Wiskott-Aldrich syndrome (WAS), metachromatic leukodystrophy (MLD), and severe lipoprotein lipase deficiency disorder (LPLD) (52, 71). In fact, the possibilities for gene therapy-mediated treatments are endless because virtually every cell in the human body is a potential target for genetic manipulation. Viruses display specificity in infecting cell types; therefore, viral vectors can be selected based on the type of cell that needs gene delivery. Here, we describe some widely used viral vectors in gene therapy.

## Adenovirus (AV)

AV was the first viral vector developed for gene therapy and was approved for clinical trials in 1990. It was isolated from human adenoid tissue-derived cell cultures for the first time in 1953, hence the term adenovirus, and included in a diverse family of non-enveloped double-stranded DNA (dsDNA) viruses called *Adenoviridae* (72). According to the Centers for Disease Control and Prevention (CDC), AV rarely causes serious illness and death in healthy individuals but immuno-compromised individuals may develop a wide range of illnesses including the common cold, sore throat, bronchitis, pneumonia, diarrhea, conjunctivitis, fever, and neurologic disease. As of today, there are 57 human AV serotypes isolated and classified into seven categories based on their properties of agglutination (73, 74). AV carries a linear dsDNA ranging from 26 to 45 kb in a medium sized (~100 nm) non-enveloped icosahedral viral particle composed of penton and hexon subunits. While the hexon subunits form a major part of the viral capsid coat and carry antigenic motifs, the penton subunits constitute fiber and knob domains required for infection (75). The fiber knob domain initiates AV infection by binding to a variety of proteins such as MHC-1 α2 subunit, CD46, sialic acid saccharides on glycoproteins, coxsackievirus, and AV receptor (CAR) expressed on cell surface (76). The interaction between arginine-glycine-aspartic acid (RGD) sequence of the fiber penton subunit and αν integrins on the cell surface drives endocytosis of viral particle and completion of viral infection (77–79). This creates broad tissue tropism and a nodal for AV transduction efficiency, giving an opportunity to manipulate binding sites for CAR and other ligands to de-target AV infection, an essential feature of popular viral vectors used in gene therapy. Therefore, since its discovery, AV has been repurposed through the deletion of its pathogenic genes.

Based on the expression of AV genes during infection and multiplication, its genome is organized into early (E1, E2a, E2b, E3, and E4), intermediate (IVA2 and IX), and late genes (L1, L2, L3, L4, and L5). Also, its genome carries non-coding inverted terminal repeat (ITR) sequences, ψ packaging sequences, and many viral RNAs (75, 80, 81). The genome of AV has been manipulated many times to develop safe and efficient vectors for gene therapy applications. The first-generation vectors with a partial deletion of E1 or E3 genes do not replicate or display oncogenicity but can deliver less than an 8 kb gene and display leaky expression of viral proteins, strong immune response, and contamination with replication-competent virus (82). To circumvent this, second-generation vectors were created by deleting E2A, E2B, and E4 from the genome of the first-generation AV vectors. However, their production has become complicated, and they do not prevent leaky expression of viral proteins and rapid loss of therapeutic gene expression, and thus have lost enthusiasm for their widespread use in gene therapy (83, 84). The third-generation vectors, otherwise known as gutless or helper-dependent AV vectors, lack all viral genes except the ψ and ITR sequences. They have received special attention because of their capacity to carry larger therapeutic genes (up to 37 kb in size), their ability to display long-term transgene expression, and lesser contamination with replicating virus particles. They are also less immunogenic than first- and second-generation vectors (85). The third-generation vectors were successfully used to express transgenes for about 2 years in animals with no adverse effects (86, 87). Co-transduction of these vectors with Sleeping Beauty transposon along with FLP recombinase was used to insert a FIX gene in the chromosome of dogs suffering from hemophilia B and expressed for up to 960 days (88, 89). Recently, they were successfully used for the long-term expression of a gene encoding an alanine-glyoxylate aminotransferase (AGT) in patients with primary hyperoxaluria type 1 (PH1), a rare kidney disorder that causes recurrent kidney stones (90).

**Figure 2:**
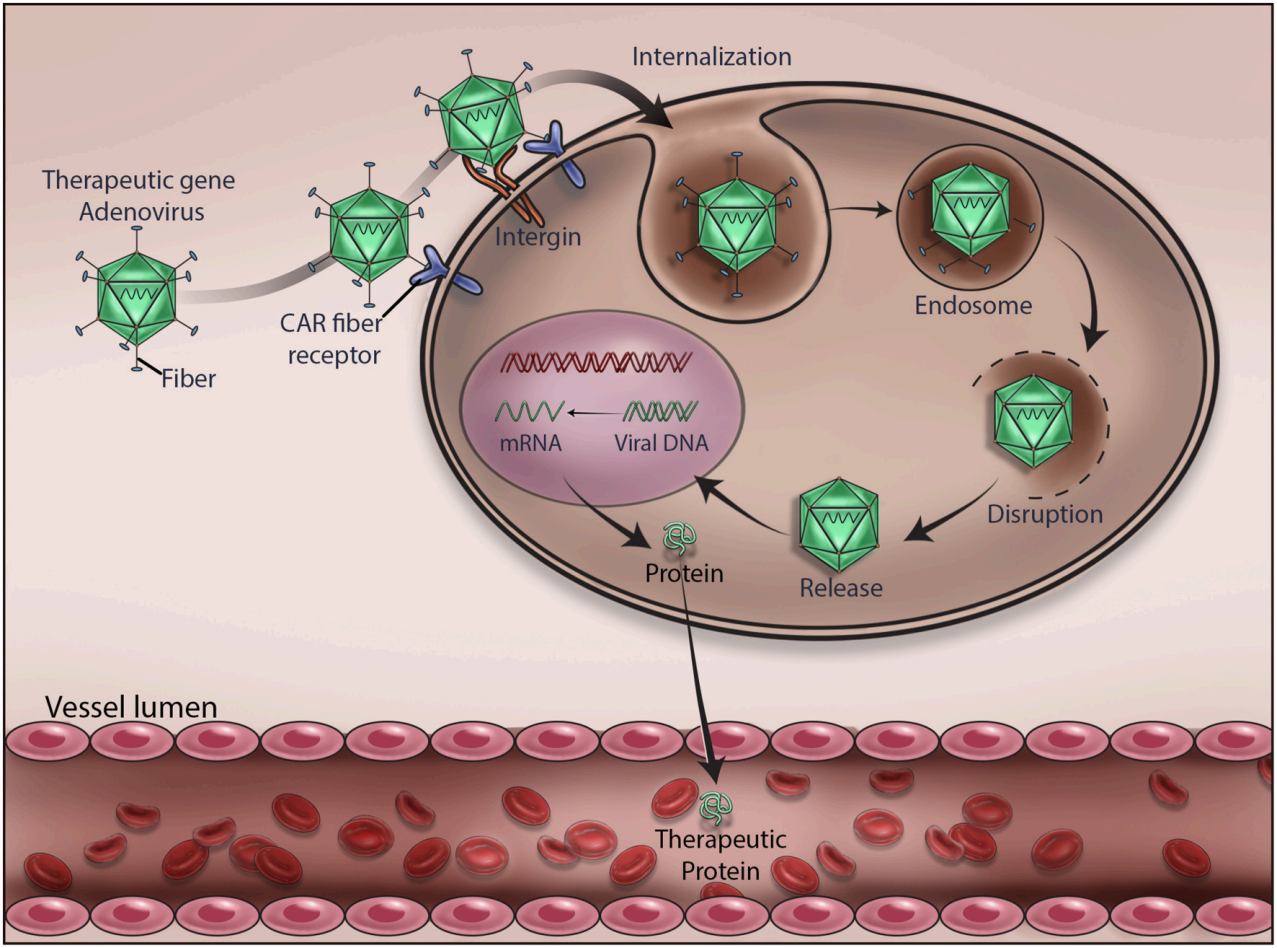
Mechanism of adenovirus-mediated delivery of a therapeutic DNA. Upon infection, adenovirus delivers the encapsulated therapeutic DNA into the cytoplasm of the target cells. Various stages of viral gene delivery, *viz* cell attachment, internalization, endocytosis, uncoating, transcription and translation of the therapeutic protein, are shown.

Since AV vectors allow episomal or stable insertion of therapeutic genes, they carry advantages over vectors that integrate into cellular DNA. This provides clinicians an opportunity to offer appropriate treatment for patients with different diseases or disorders. For instance, AV is suitable for treating cancers and offering bioscavenger-mediated short-term protection against nerve gases and other chemical weapons. As depicted in Figure 2, we have demonstrated an AV-mediated episomal insertion of *PON1, BChE*, and *PD* bioscavenger genes in the liver to express and secrete proteins to detoxify the circulating lethal nerve gases for 10–15 days in mice (13, 20–22, 36). Since the immune system has the natural ability to detect and destroy abnormal cells in our body, new AV vectors that can induce immune response and destroy target cells have been developed. For example cancer cells can go undetected by reducing the expression of tumor antigens on their surfaces, inducing immune cell inactivation, and releasing substances in the microenvironment to promote their growth and survival. Therefore, new oncolytic adenoviruses that effectively induce immune response, and specifically target and lyse tumor cells are being created by replacing their native E1A promoter with tumor-specific promoters and genetically modified CAR, a highly expressed AV receptor in tumor cells (68, 91). For example, the CV706 and OBP-30 AV vectors carry the viral E1A gene under prostate cancer-specific antigen promoter and telomerase reverse transcriptase promoter, respectively (92). Other engineered oncolytic adenoviruses target the components of tumor cells and their microenvironment and inhibit their proliferation by expressing antibodies, relaxin, hyaluronidase, and inhibitors of metalloproteinases to hinder angiogenesis and proper function of the extracellular matrix (91, 93). Oncolytic adenoviral vectors that induce autophagy-related immunogenic cell death were also developed to treat cancer (94). A novel oncolytic AV vector expressing an interfering long non-coding RNA (lncRNA) to inhibit 12 oncogenic miRNAs has been constructed in order to perform selective killing of tumor cells (95). AV vectors carrying complementary sequences of liver-specific miRNA-122a incorporated into 3'-UTR of *E2A, E4*, or *pIX* to reduce the leaky expression of viral genes and hepatotoxicity were developed (96). In addition, AV vectors with *E1A* carrying mutations complementary to retinoblastoma (RB) or p53 gene mutations in tumor cells that can specifically replicate and lyse tumor cells were created (92).

Despite many technological improvements made in vector design and production, there are still certain issues that have to be addressed for better clinical outcome. For example, infecting target cells with the optimal amount of highly purified AV particles is critical for the successful insertion of a therapeutic gene. Recently, it was shown that 10^10^-10^12^ AV particles per patient are required for a successful Ebola vaccination (97). Production of such high titer virus with no or minimal empty vector contamination is still a formidable challenge. Also, high prevalence of anti-AV vector immunity in the human population and differential expression of CAR and other receptor proteins on target cells have been serious issues in clinical trials (98, 99). For example, the transduction efficiency of the widely used AV serotype 5 in gene therapy is dampened by the prevalence of neutralizing antibodies in the human population (100, 101). An estimated 80% of the human population is believed to carry antibodies against AV serotype 5, resulting in a significant transduction deficiency and stimulation of inflammatory shock (102). There has been a positive correlation between body fat and the presence of circulating antibodies against AV serotype 36 in humans (103). In addition, during systemic administration, the tendency of AV vectors to undergo sequestration in the liver has prevented efficient transgene transduction and displayed severe hepatotoxicity, even causing the death of a clinical trial participant (104, 105). This was due to the binding of blood coagulation factor FV and FX to the hyper variable region (HPV) of AV hexon subunit (106, 107). Therefore, mutating the HPV site in such a way that it neither activates complementary pathway nor interacts with FX could be an ideal way to resolve the liver sequestration issue. Attempts are being made to improve the safety of AV vectors by treating with chemicals and developing chimeric and hybrid vectors to minimize inflammation and immunogenicity (108, 109). For example, the chimeric AV serotype 5 vector carrying receptor-binding epitopes derived from other human AV serotype 3, serotype 35, and serotype 43 displayed low seroprevalence and low affinity for CAR (110, 111). Similarly, the chimeric human AV serotype 5/3, consisting of receptor epitopes derived from serotype 3 and 5, showed high binding affinity for CD46, an AV receptor commonly expressed on many solid tumors. It was thus found to be particularly useful in targeting solid tumors (112, 113). Another CD46-targeted chimeric AV vector derived from human serotype 5 and 35 has been shown to be suitable for transducing vascular smooth muscle cells, treating colorectal cancers, and ischemic wounds as well as manipulating T-cells (114–117). Novel chimeric AV vectors developed from AV serotypes 5 and 11 and 3 and 11 were found very effective in exclusively targeting glioma and colon cancer cells, respectively (118, 119). Other types of chimeric vectors were also derived entirely from low prevalent human and non-human AV serotypes such as human AV serotype 26, canine AV serotype 2, and chimpanzee serotype 3. For example, the chimeric AV vector developed from human AV serotype 26 and chimpanzee AV serotype 5 has been used successfully for Ebola vaccination in animal models (120, 121). A novel hybrid vector developed from AV serotype 5 and alpha virus was found very useful for the expression of transgenes in malignant hematopoietic cells (122). Many laboratories have developed a library of AV vectors that carry random-peptides on their fiber knobs in order to overcome the paucity of cancer-specific ligands (123–125). This resulted in the generation of many AV vectors that are specific to prostate and pancreatic cancer as well as glioma (123, 125–128). One such vector carrying pancreatic cancer-targeting ligand has shown strong oncolytic effect in primary pancreatic neuroendocrine tumors and appears promising as a next-generation therapy (129). Given the advancements made in developing safe and efficient AV vectors, their choice for delivering therapeutic genes has become apparent in clinical trials.

## Adeno-Associated Virus (AAV)

AAV is yet another popular viral vector used in gene therapy. This small microbe was first isolated as a contaminant in the simian adenovirus preparation and then named adeno-associated virus (AAV) by the Bob Atchison group at Pittsburgh University and the Wallace Rowe group at the NIH (130, 131) and later found in a wide range of animal samples including human, non-human primates, avian, bovine, reptiles, pigs, sea lions, bats, and caprine samples. The 4.7-kb-long single-stranded DNA (ssDNA) packed inside a non-enveloped viral particle carries p5, p19, and p40 promoters as well as rep and cap genes flanked by two 145 nucleotide-long inverted terminal repeats (ITR) and no polymerase gene (132, 133). While ITRs having palindromic sequences base pair to allow synthesis of cDNA, both rep and cap genes undergo alternate splicing to express replication proteins (Rep78, Rep68, Rep52, and Rep40), capsid or virion proteins (VP1, VP2, and VP3), and an assembly activating protein (AAP), respectively (134). Being a non-structural protein, AAP assists virion proteins in capsid formation (135). VP1, VP2, and VP3 expressed from p40 promoter at a ratio of 1:1:10 form the outer capsid of the virion. These capsid proteins carry phospholipase domain to protect virions from the onslaught of intracellular protease system (136). Unlike other viruses, AAV requires a few other helper proteins, agents or viruses such as AV, herpes simplex virus type I/II, pseudorabies virus, cytomegalovirus, genotoxic agents, UV radiation, or hydroxyurea to infect cells and complete replication (137). AAV can also be generated by providing the missing genes E1a, E1b, E2a, E4orf6, and VA that are needed for viral infection. These genes are often cloned in pXX6 helper plasmid and used to co-transfect HEK293 cells along with AAV expression plasmid (rep-cap plasmid) to produce AAV (134, 138). Therapeutic genes are cloned in the AAV expression plasmid carrying ITR sequences, and their size can be increased by cotransfecting another plasmid carrying rep-cap genes or by generating virus in rep-cap stable cells. Since the formation of dsDNA from its ssDNA is the rate-limiting step of viral infection, gene delivery, and expression in the target cells, a self-complementary viral dsDNA (scAAV) is developed; however, it reduces the capacity of AAV vectors to deliver a therapeutic gene (139, 140). AAV inserts a therapeutic gene in the genome of target cells to provide long-term transgene expression. For instance, the gene expressing FIX blood coagulation factor in one individual of a cohort persisted for more than 10 years during a clinical trial (141). AAV inserts a therapeutic gene in the host genome at a specific location on the q arm of chromosome 19 (142, 143). Despite having no large homology regions, more than 70% of the transgene integration events occur within this site; however, the underlying mechanism remains unknown. But AAV lacking its rep-cap genes can deliver a therapeutic gene in the episomal form without inserting into the genome of the target cells. The therapeutic gene in the episomal form can develop into a chromatin-like structure and remain quiescent in cells for months to years without damaging the patient's body. Recently, we have used AAV vectors to make episomal insertion and expression of a bioscavenger gene in the liver cells for about 6 months (unpublished results). This allows clinicians to apply AAV-mediated gene therapy to treat a wide variety of diseases or disorders.

AAV displays broad tropism but requires the expression of heparin sulfate proteoglycan, α_v_β_5_ integrin, α_5_β_1_ integrin, fibroblast growth factor receptor 1, platelet-derived growth factor receptor, hepatocyte growth factor receptor, epidermal growth factor receptor, laminin receptor, and sialic acid moieties on the surface of target cells for efficient transduction and delivery of a therapeutic gene. Recently, AAVR has been identified as a universal host cell receptor for AAV infections (144). Although every serotype has the ability to infect cells, transport to nucleus, uncoat, and insert its genome into the host's chromosome or leave in the episomal form, not all 13 AAV serotypes isolated to date use the same receptor repertoire on host cell surface for infection (145, 146). This makes AAV a very useful system for a specific cell or tissue type transduction. For example, AAV1 displays high transduction efficiency of muscles, neurons, heart, and retinal pigment epithelium. AAV2 has been shown to infect many types of cancer cells, neurons, kidney, retinal pigment epithelium, and photoreceptor cells. AAV2 is the only serotype that can infect and delivery a therapeutic gene to kidney. AAV4 and AAV5 serotypes infect retina and retinal pigment epithelium, respectively. While AAV6 displays strong tropism for heart, AAV7 has some bias for liver (147). AAV6 is also effective in infecting airway epithelial cells (148). AAV8 and AAV9 have displayed successful infection of lymphoma and HPV tumors, respectively (149). AAV8 is the only serotype that infects pancreas, and it was extensively used to express a therapeutic *FIX* in the liver to treat hemophilia in clinical trials (150). AAV tropism was further refined by mixing the capsid proteins of one serotype with the genome of another serotype. For example, AAV2/5 serotype, which transduces neurons more efficiently than the parental AAV2, was generated by packaging AAV2 genome in AAV5 capsid proteins. Another example, the pseudotyped AAV8 and AAV9, can cross the endothelial barrier of blood vessels to target muscles (66). For increasing transduction efficiency, hybrid AAV serotypes were also generated by mixing the capsid proteins of multiple serotypes with the genome of another serotype. For example, AAV-DJ serotype that consists of a hybrid capsid is generated by mixing the capsid proteins of eight different AAV serotypes. This made AAV-DJ to display higher transduction efficiency than any other wild type serotype *in vitro* and high infectivity of a broad range of tissue *in vivo*. Its mutant, AAV-DJ8 serotype, displays high infectivity of brain. AAVHSC, a new class of genetic vector isolated from hematopoietic stem cells, has been shown to be ideal for manipulating stem cells (151). Since more than 50% of the adult human population carries AAV neutralizing antibodies, a wide range of mosaic or hybrid vectors were generated by engineering and *de novo* shuffling of capsid proteins (152, 153). For example, the AAV2.5 hybrid vector generated by combining the muscle tropism determinants of AAV1 with parental AAV2 displays immune evasion of their neutralizing antibodies (154). The other hybrid vectors AAV6.2, AAV2i8, AAVrh10, and AAVrh32.33 were found beneficial for intravenous delivery, reduction of liver sequestration, and T-cell response in the clinic, respectively (138, 155–157). Since CpG motifs are responsible for immune response and failure of many clinical trials, AAV vectors were further refined by deleting CpG motifs, known ligands of Toll-like receptor 9 (TLR9), to reduce immune response for maximal expression of a transgene in clinical trials (158). Cre-recombination-based AAV variants are also developed to allow efficient transgene expression in the central nervous system, muscle, and liver (159, 160). Also, the AAV-CRISPR/Cas9 system has been developed to perform *in vivo* genome editing and broaden therapeutic horizons (161).

## Herpes Simplex Virus (HSV)

Herpes simplex viruses are believed to have tremendous potential as a preventative and therapeutic vaccine against cancer and other diseases because of their ability to evade the immune system and circulating anti-viral drugs, deliver multiple genes, infect a wide variety of cells, pose low risk of adverse health effects, and multiply specifically in tumor cells. They are large enveloped viruses that carry a linear dsDNA of 120–240 kb and infect reptiles, birds, fish, amphibians, and mammals. There are eight known human herpesviruses: herpes simplex virus-1 (HSV-1), herpes simplex virus-2 (HSV-2), human cytomegalovirus (HCMV), varicella-zoster virus (VZV), Epstein-Barr virus (EBV), Kaposi's sarcoma-associated herpesvirus (KSHV), and human herpesviruses 6 and 7 grouped under alpha, beta, and gamma genera. Though they share common virion structure and replication cycle, and many other biological properties, there is a significant difference in their tropism, infection, and clinical manifestations. Some of their genes show homology with regions of human chromosomes. Here, we delve into HSV that infects ~60% of the human population worldwide and mainly transmits through contact, especially oral-oral contact (162). After infecting oral or genital epithelial cells, HSV enters neurons to establish lifelong latent infection and reactivates periodically causing fever, blisters, cold sores, genital herpes stromal keratitis, blindness, cancer, and encephalitis. Both HSV-1 and HSV-2 carry envelope and sub-envelope structures called tegument and a regular icosahedral capsid consisting of a relatively large dsDNA of 153 kb encoding ~200 proteins (163). Nearly half of the total 84 genes present in the HSV genome encode proteins required for virus replication, and many were found unnecessary for delivering therapeutic genes. Several genes involved in virulence and immune evasion, and those considered non-essential for viral life cycle *in vivo* were also identified. HSV-1 is relatively less pathogenic than HSV-2 and is, therefore, ideal for vector development and gene therapy (164). HSV infects cells by using its envelope glycoprotein B, glycoprotein C, and glycoprotein D to bind cell surface particles and transmembrane receptors such as heparin sulfate, herpesvirus entry mediator (HVEM), nectin-1, and 3-O sulfated heparin sulfate. While the nectin receptors provide a strong point of viral interaction with the host cell, the other viral envelope proteins, especially glycoprotein B, glycoprotein D, glycoprotein H, glycoprotein L, and HVEM, create an entry pore for the viral capsid. The viral capsid enters through capsid pore and travels through the cytoplasm to the nucleus in order to inject its DNA content. HSV evades the immune system by secreting its immediate-early protein, ICP47, and inducing a transporter associated antigen processing (TAP) protein to block MHC class I antigen presentation on the cell surface. HSV-1 infects many types of mitotic and post-mitotic cells including neurons (36). After infection, HSV induces the expression of the virion host shutoff protein (VHS or UL41) to inhibit protein synthesis by degrading the host mRNA. This makes way for viral replication and lysis of the host cells.

The HSV genome carries immediate-early, early, and late genes for replication and allows creation of replication-competent, replication-incompetent, and helper-dependent vectors, or amplicon vectors, for preclinical and clinical studies. The replication-competent vectors have the capacity to deliver transgenes up to 50 kb in size or the entire locus since treatment of certain diseases requires huge therapeutic gene cassettes carrying complex regulatory elements. These vectors can replicate selectively in cancer cells and have less virulence because of deleted genes. They do not insert transgenes in the host chromosomes and are therefore used primarily as oncolytic viruses to treat glioma, melanoma and ovarian cancers and to stimulate an immune response (66). They are further refined by using tumor specific promoters to express viral genes and target tumor-specific receptors. These vectors with robust replication capacity are believed to enhance intramural vector distribution and lyse tumor cells very effectively. These vectors are generally constructed by homologous recombination in eukaryotic cells by co-transfecting the viral genome and a plasmid carrying the therapeutic gene flanked by the sequences homologous to the target locus on viral genome to undergo recombination. The replication-incompetent vectors are created by either mutating or deleting several immediate early genes including ICP4 and ICP27 that are essential for replication and, therefore, can grow only in specifically designed cell lines complemented transiently. For example, Vero-7b cell line is capable of providing in trans the proteins encoded by deleted viral genes (165). They are safe and non-inflammatory advanced vector platforms known to persist and express in the nerve cells for life and therefore used to treat neuropathic, inflammatory, and cancer-associated pain (166–168). The helper-dependent HSV vectors, or amplicon vectors, carry deletions in one or more non-essential genes and retain the ability to replicate *in vitro* but are compromised *in vivo* in a context-dependent manner (169, 170). These viruses are the same as wild type HSV, with plasmids containing a packaging signal and the gene of interest. Amplicons have the ability to accommodate a very large therapeutic sequence up to 100 kb in size but have production and stability issues. The replication-incompetent vectors and amplicons have been used to express genes in the nervous system, muscle, heart, and liver.

## Retrovirus (RV)

Retroviruses are spherical (~100 nm in diameter) and enveloped microbes belonging to a *Retroviridae* family that comprises foamy virus, human immunodeficiency virus (HIV-1), simian immunodeficiency virus (SIV), bovine immunodeficiency virus, feline immunodeficiency virus, equine infectious anemia virus (EIAV), murine leukemia virus (MLV), bovine leukemia virus, Rous sarcoma virus (RSV), spleen necrosis virus (SNV), and mouse mammary tumor virus. Unlike all other RNA viruses, these viruses are capable of reverse transcribing their genetic blueprint of positive, single-stranded RNA into dsDNA, and inserting it into the host cell genome. RVs carry a non-covalently linked dimers (two copies) of RNA genetic material probably as a fail-safe mechanism for producing genomic DNA and increasing viral RNA diversity due to interstrand recombination (171). Thus, RNA dimerization has been viewed as a prerequisite for RV genome encapsidation and life cycle. With restricted vertebrate hosts, these viruses are divided into exogenous retroviruses (XRV) and endogenous retroviruses (ERV). While the XRVs transmit horizontally among hosts, the ERVs inherit vertically in the genome of their hosts (172). By scattering all over chromosomes and comprising nearly eight percent of the human genome, the ERVs are thought to be relics of ancient retroviral germline infections and believed to play a role of friend or foe in human life (173–175). The two most common types of retroviruses are gammaretrovirus and lentivirus, which are derived from MLV and HIV-1, respectively. The genome of gammaretrovirus has three essential genes, *gag, pol* and *env*, and is flanked on both sides by long terminal repeats (LTRs). *Gag* inserts viral genome mRNA into virions when assembling, *pol* is the reverse transcriptase, integrase, and protease encoding gene, and *env encodes the surface and the transmembrane glycoprotein*. Also, RV genome carries a cis-acting ψ packaging element that involves in regulating the essential process of packaging the RV mRNA into viral capsid during replication. In addition, RV genome carries RNA dimerization signal element. With U3, R, U5 elements, the LTRs display promoter/enhancer activity for gene transcriptional regulation. RVs use their envelope proteins to bind a variety of receptor molecules such as murine cationic amino acid transporter (mCAT), a sodium-dependent Pi transporter (PiT2), xenotropic and polytropic retrovirus receptor 1 (XPR1), CD4, CD46, CD150, and the RD114-and-D-type-retrovirus/alanine-serine-cysteine transporter 2 (RDR/ASCT2) expressed on different cell surfaces to initiate infection, a critical step in determining the target cell tropism of the virus. This leads to a conformational change in the envelope proteins, leading to the entry of virus into the cytoplasm via fusion or endocytosis. With the help of the host cell proteins, the endosome travels through cytoplasm to eject its RNA. After the RNA reverse transcription takes place, viral DNA is integrated into the host cell chromatin, transcribed into RNA with 5' Cap and 3' poly(A) tail, and translated into viral proteins that assemble and bud from the plasma membrane to complete the life cycle with extracellular maturation (171). The matured RVs can infect a wide variety of somatic cells including embryonic stem cells, hematopoietic and neural stem cells. With active nuclear elements, these vectors can transduce therapeutic genes into proliferating cells only and are, therefore, ideal for targeting specifically cancer cells. A downside to gammaretrovirus is that it has broad species specificity, leaving the possibility of transducing undesired cells, faulty reverse transcription, intracellular restriction factors, and risk of insertional mutagenesis. The major difference between gammaretrovirus and lentivirus is that lentivirus can infect post-mitotic cells. It requires four plasmids for production: the *gag* and *pol* plasmid, the *rev* plasmid to transport mRNA into the cytoplasm, VSV-G for membrane fusion and the gene of interest. Other retroviruses require three plasmids: the *gag* and *pol* plasmid, the VSV-G plasmid and the gene of interest. Transient or stable co-expression of all these plasmids in HEK293T packaging cell lines produces RV vector particles carrying no replication-competent virions that are essential for research and therapeutic purposes. Using these cell lines, methods to produce clinical grade RV particles at a concentration of 10^6^ to10^7^/ml are optimized (176). As *gag*/*pol* and *env* expression constructs carry no ψ packaging and RNA dimerization element, viral structural proteins only recognize the ψ-containing RV vector construct resulting in a preferential packaging of RV vector genomes into infectious particles. After entry of the particle into the host cell, only the RNA of the RV vector construct is reverse transcribed and stably integrated into the host genome. This method prevents generation of replication competent retroviral vector progeny during therapeutic viral particles production. Lentivirus has been used to treat X-SCID, cancers and monogenic diseases. For example, self-inactivating lentiviral vectors can engineer T cells with receptors to better target tumors when treating cancer. Recently, we have successfully used lentiviral vectors to deliver an anti-angiogenic Kininogen gene to budding blood vessels (177, 178). There have been no reports of significant adverse effects from the lentivirus (37).

Some advantages of using retroviruses are that they can accommodate a 9–12-kb-large insert size for the gene of interest and produce high titers. The most significant disadvantages are lack of cell specificity and the possibility of insertional mutagenesis (18). The enzyme “integrase” inserts copies of the retroviral genome into the host cell chromosomes but there is a risk of inserting the genome copy into an unfavorable location such as a tumor suppressor gene or an oncogene, which would lead to uncontrolled cell division (36). It is critical to evaluate the risk of insertional mutagenesis for each retroviral vector. Gammaretroviral vectors have a tendency to integrate near gene regulatory regions, which can pose a significant risk. For example, patients in a cohort of 20 died due to leucosis development in a clinical trial (179). On the other hand, lentiviral vectors tend to integrate into the body of genes, leading to lower risk of genotoxicity (52). A possible step to address this issue would be to use self-inactivating retroviral vectors that are transcriptionally inactive. Since mature T cells are relatively resistant to oncogenic transformation by RV, developing T cell-based therapeutic approaches to treat cancer and other diseases would be another approach to avoid insertional mutagenesis. Recently, a non-integrating RV-based CRISPR/Cas9 vectors have been created for targeted gene knockout (180). Creating such vectors to target specific genes would help developing therapeutic approaches without insertional mutagenesis issue. Renal fibrosis was treated by using high-fidelity RV-based CRISPR/Cas9 vectors (181). Development of similar vectors would not only address insertional mutagenesis issue but also radically transform basic and applied biomedical research. Also, using AAV vector which inserts a therapeutic gene selectively into known chromosome 19 sequences would be another possibility. Using zinc finger nucleases or including certain sequences such as the β-globin locus control region to direct the site of integration to specific chromosomal sites is yet another way to minimize the risks. However, further studies are needed to address this issue by designing specific vectors and understanding the frequency of insertional mutagenesis, and role of other factors involved. Insertional mutagenesis is an issue that will likely be solved in the coming years. Until then, the use of retroviruses remains a concern. Nonetheless, over 500 gene therapy clinical trials have been conducted using retrovirus to date.

## Gene Therapy Drugs in the Market

**Figure 3:**
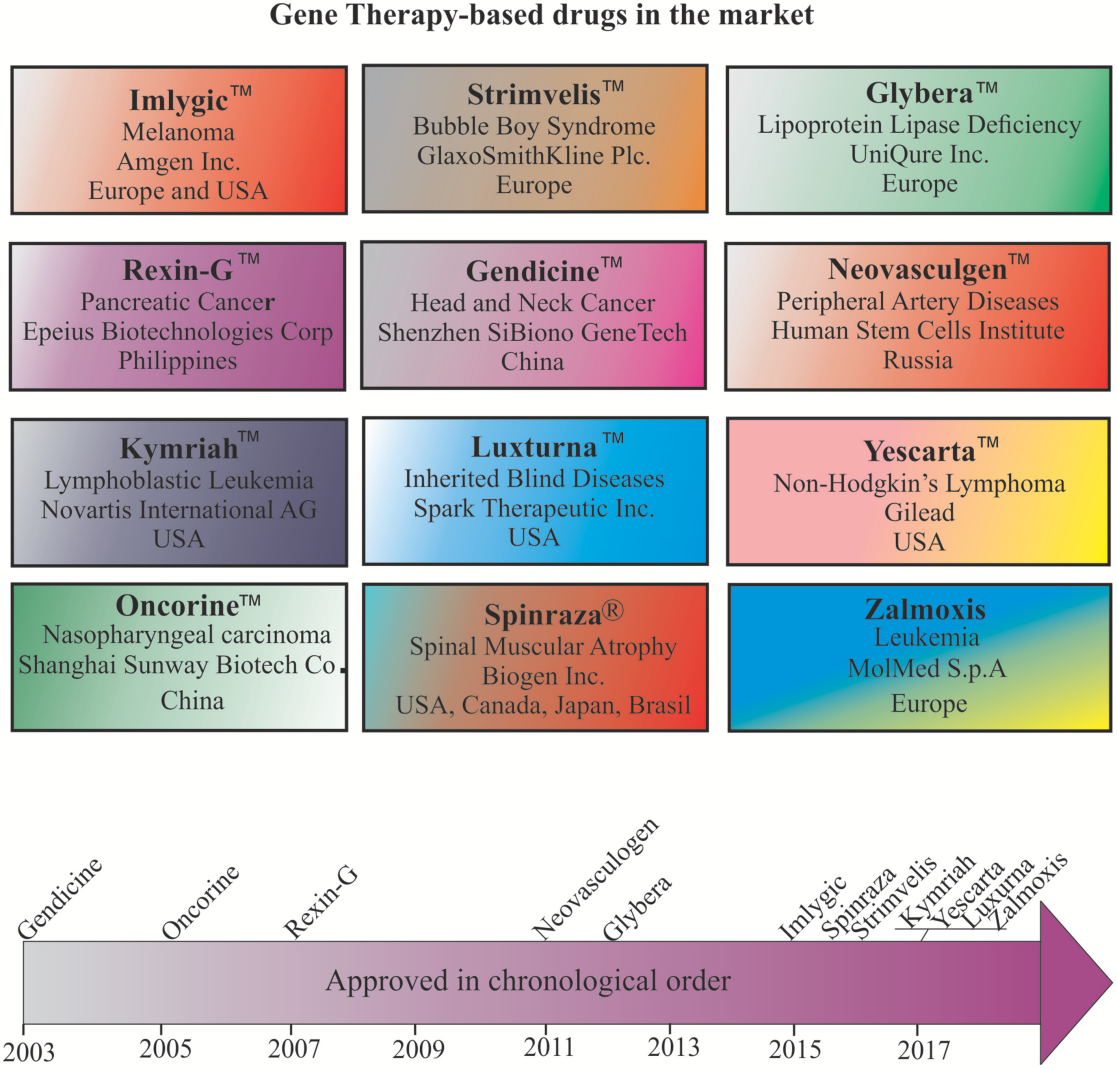
Gene therapy drugs in the pharmaceutical market and a timeline of their approval.

Despite many technological challenges and barriers, more than a dozen gene therapy-based drugs have entered the world pharmaceutical market to date (Figure 3). The first gene therapy drug, Gendicine™, was developed by Shenzhen SiBiono GeneTech for the treatment of patients with tumors carrying a mutated p53 gene, a common cause for more than 50% of all types of human cancers. The State Food Drug Administration of China approved Gendicine™ for the treatment of head and neck squamous cell carcinoma on October 16, 2003 (182, 183). However, the USFDA has turned down Introgen's Advexin, another AV-based viral drug that uses p53 due to concerns about the safety of the AV vectors after Jesse Gelsinger died in 1999 while participating in a clinical trial but no information is available about the submission of Gendicine™ clinical data for approval from the USFDA to date. Gendicine™, a replication defective AV loaded with wild-type p53 gene, is given to patients by less invasive intramural injections and or intracavity infusions. According to the manufacturer, a single dose of this viral drug, costing less than US $400, is given to patients once a week for 8 weeks as a cure. After injection, the therapeutic activity of p53 activated by target tissue cellular stress induces cell-cycle arrest, DNA repair, apoptosis, senescence, and autophagy to cause tumor growth regression. Gendicine™ has been given to more than 30,000 cancer patients, and it has displayed an exemplary safety record with no significant side effects to date (14). According to the manufacturer, Gendicine™ has shown a higher response rate when combined with chemotherapy and radiotherapy in comparison with standard therapies alone. Because Gendicine™ is injected directly into tumors and becomes useless for treating tumors neither detectable nor accessible, other advanced replication-defective AV-based drugs, such as Advexin and SCH-58500, that carry wild type p53 gene were developed to target all tumors in the patient's body in an intravenous injection; however, neither Advexin and SCH-58500 has entered the pharmaceutical market to date. However, Oncorine™, another replication defective AV-based drug that carries p53 gene to cure head and neck cancer, made it to the Chinese pharmaceutical market in 2005. According to the manufacturer, Shanghai Sunway Biotech Co., the curative effective of Oncorine™ combined with chemotherapy is superior to chemotherapy alone with a good safety profile. Since low transduction is a major issue with these approved replication defective AV drugs, more advanced tumor-specific p53-expressing conditionally replicating AV vectors such as ONYX 015, AdDelta24-p53, SG600-p53, H101, and OBP-702 have been developed but none of them is approved for cancer treatment to date. As many clinicians prefer cancer management rather than a cure due to the complex nature of the disease, the future of oncolytic viral therapy demands further advancement in vector design and discovery of appropriate therapeutic genes for better treatment.

The next advanced gene therapy drug, Rexin-G™, a chimeric retrovector loaded with a cytocidal dominant negative cyclin G1 gene to target and kill solid tumors, was approved by the Philippines FDA in 2005. Rexin-G™ developed by Epeius Biotechnologies Corporation was designated by the US FDA as an orphan drug for pancreatic cancer. After intravenous injection, this viral drug carrying a motif derived from von Willebrand coagulation factor (vWF) on its surface selectively binds receptors and collagenous proteins exposed heavily in tumor microenvironment in order to fuse, enter, uncoat, and insert its genetic material randomly in the chromosomes of the actively dividing tumor cells only (184). Recent clinical studies confirmed its safety, anti-tumor activity, and potential to increase survival time and survival rate of patients. Recently, another retrovirus-based drug, Strimvelis™, was approved in Europe to treat an ultra-rare immunodeficiency syndrome, ADA-SCID, or Bubble Boy Syndrome, a fatal and life-threatening disease due to lymphopenia, and recurrent and opportunistic infections. A bone marrow transplant from a young child donor with matched leukocyte antigen is the recommended treatment for ADA-SCID patients, but the availability of a suitable donor is challenging. Therefore, Strimvelis™ is designed and developed to offer *ex vivo* gene therapy and involves use of RV to insert copies of the ADA gene into the chromosomes of stem cells extracted from the bone marrow of patients. The stem cells carrying the ADA gene are then reintroduced into the patients whose bodies can express protein to repair their immune system on their own. This drug, with a list price of $714,000, is available for ADA-SCID patients without a donor that has matched human leukocyte antigen (HLA). Clinical studies revealed a 100% remission rate for Strimvelis™ (Table 2). Nonetheless, there is now a push toward using self-inactivating retroviral vectors that have less risk of insertional mutagenesis, especially self-inactivating HIV-1-based lentiviral vectors (185). A few months ago, the FDA approved Kymriah™, a lentivirus-based chimeric antigen receptor T cell (CAR-T) therapy for acute lymphoblastic leukemia (186). The underlying mechanism of this cancer type disease development still remains unknown, but patients carry abnormal lymphocytes in many of their body parts. Kymriah™ was developed by Novartis in collaboration with the University of Pennsylvania to treat patients with non-Hodgkin lymphoma (NHL) and B-cell acute lymphoblastic leukemia (ALL). Kymriah™ is a novel immunocellular therapy that uses a patient's own reprogrammed T cells with a transgene encoding CAR to identify and eliminate CD19-expressing malignant and non-malignant cells; overall remission rate with the therapy is 83% (Table 2). The autologous peripheral blood T cells are reprogrammed to carry intracellular 4-1BB and CD3-zeta costimulatory domains fused with a murine single-chain antibody fragment in its CAR to recognize CD19 increase, cellular expansion, and persistence. Yescarta™ is another retrovirus-based CAR-T cell immunotherapy developed by Kite, a Gilead company, and approved by the FDA in 2017. This breakthrough hematologic cancer drug is a customized treatment generated using an NHL patient's own T-cells to help fight lymphoma. The patient's T-cells are collected and genetically modified using a RV to generate a CAR consisting of anti-CD19 CAR-T cells linked to CD28 and CD3-zeta co-stimulatory domains. This drug is specifically designed to treat diffuse large B-cell lymphoma (DLBCL), a common aggressive NHL that accounts for three out of every five cases. According to the manufacturer, ~7,500 patients with refractory DLBCL are qualified to receive Yescarta treatment in the USA alone. With a list price of $373,000 in the USA, Yescarta is believed to get approval for the European market in the near future. Zalmoxis is another T-cell based medicine designated an orphan drug and approved by the EMA for treating certain leukaemias and lymphomas. This is used as an add-on treatment in patients who received hematopoietic a stem cell transplant (HSCT) from a partially matched donor to restore the immune system. This is a somatic cell therapy product consisting of T-cells that are genetically modified using a RV to express ΔNGFR and HSV-TK Mut2 suicide genes. This drug sometimes attacks the patient's body by causing graft-vs.-host disease, but the suicide gene makes these T cells become susceptible to ganciclovir or valganciclovir medicine commonly given to treat and prevent further disease development.

Neovasculgen™, a non-viral first-in-class gene therapy drug developed by the Russian Human Stem Cells Institute, has been available since 2012 for the treatment of atherosclerotic peripheral arterial disease (PAD) including critical limb ischemia. Intramuscular injection of a single dose of this drug, costing less than $50, delivers a plasmid DNA-carrying VEGF gene cloned under a CMV promoter and stimulates angiogenesis and blood supply to decrease the risk of amputation and death in patients suffering from PAD. A recent post-marketing surveillance study revealed a significant increase in pain-free walking distance by PAD patients and confirmed the therapeutic efficacy of this drug (56, 57). Recently, Spinraza® has become the first approved treatment for the rare and often fatal disease spinal muscular atrophy (SMA). SMA patients suffer muscle strength affecting their ability to sit, stand, and breathe. SMA is caused by widespread splicing defects due to mutations in survival motor neuron 1 (*SMN*1), a ubiquitously expressed cytoplasmic and nuclear protein involved in transcriptional regulation, biogenesis of small ribonucleoproteins, telomerase regeneration, and intracellular trafficking. Although the SMA patients carry its paralog *SMN*2, low-level expression due to alternative pre-mRNA splicing appears responsible for this disease development. Therefore, Spinraza carrying *SMN*2-directed antisense oligonucleotides is designed and developed to resurrect normal SMN2 protein expression in SMA patients. This non-viral drug developed by Biogen Inc. has received orphan drug status and was approved for treating all types of SMA in the USA, Canada, Japan, the European Union, Switzerland, Australia, South Korea, Chile, and Brazil. Spinraza solution upon intravenous and or intrathecal administration enters many cells in the body and induces SMN2 protein expression. According to the manufacturer, this medicine, with a list price of $125,000 per injection, costs $750,000 per year for the first year and hundreds of thousands of dollars every year for the rest of patient's life. An AAV-mediated drug designed to express SMN1 protein in patients was developed by a Novartis company, AveXis Inc., and may become available for the treatment of SMA in the near future.

The first AAV1-based drug, Alipogene tiparvovec, or Glybera™, was approved by the EMA to treat LPLD, a rare monogenic genetic disorder characterized by accumulation of triglycerides in plasma due to mutations in *LPL*. Glybera™ carrying correct copies of *LPL* was developed by UniQure Inc., and widely heralded as the “the first gene therapy” in the Western world (Figure 3). However, only one or two people in every one million are estimated to carry LPLD, and despite Glybera' s demonstrated potential in curing LPLD, it was withdrawn from the market due to low patient demand. Recently, another AAV-based drug has entered the pharmaceutical market to treat Leber congenital amaurosis, an inherited visual dysfunction characterized by pigmented retina, wandering nystagmus, and amaurotic pupils and caused by a mutation in the *RPE65* (187). Upon completion of the late-stage clinical trials, this AAV2-based voretigene neparvovec, Luxturna™, has been designated by the FDA as a breakthrough therapy and an orphan drug for the treatment of choroideremia. Clinical trials revealed a remarkable improvement in the patients' ability to see in dim light (188). According to the manufacturer, Spark Therapeutics, Inc., Philadelphia, USA, Luxturna™ has successfully cured one blind America's Got Talent semifinalist, Christian Guardino. Recently, Luxturna™ has become the first viral-based drug approved by the FDA to treat blindness. Luxturna™, loaded with wildtype *RPE65*, will be given to patients with confirmed biallelic *RPE65* mutation-associated retinal dystrophy to restore their vision within a few months. Since Luxturna™ comes with a record sticker price, the manufacturing company offers an outcome-based rebate arrangement with a long-term durability measure and payment option over multiple years. Another AAV-based drug is poised to enter the pharmaceutical market in the near future to treat choroideremia, an X-linked inherited retinal dystrophy that causes night blindness and a constricted visual field.

Recently, the USFDA approved an HSV-based drug called T-VEC (Imlygic™) Talimogene Laherparepvec, developed by BioVex Inc., and now acquired by Amgen for melanoma treatment. T-VEC directly kills metastatic melanoma cells and enhances the immune response against them. According to the manufacturer, this advanced oncolytic virus replicates in the tumor cells and synthesizes granulocyte-macrophage colony stimulating factor (GM-CSF), resulting in tumor-lysis and release of tumor antigen, which can then trigger an immune response. The target areas include cutaneous, subcutaneous and nodal lesions. Imlygic™ also serves as an *in-situ* vaccine (189). The T-VEC treatment course involves a series of HSV injections into the melanoma lesions for 6 months for a complete cure. T-VEC was approved also in Europe and Australia for melanoma treatment. G47Δ or DS-1647 is a third generation oncolytic HSV developed by Daiichi-Sankyo Ltd., Japan, and Professor Tomoki Todo at the University of Tokyo for the treatment of malignant glioma. This has shown excellent safety and efficacy in treating glioma in preclinical and clinical studies and has been designated as an orphan drug and “Sakigake,” or ahead of the world, by the Ministry of Health, Labor and Welfare of Japan (190). However, this drug is not available for the treatment of cancer patients to date. In addition, a few more drugs are available in the market for treating different diseases (Table 2).

## Gene Therapy Drugs in Clinical Trials

**Figure 4:**
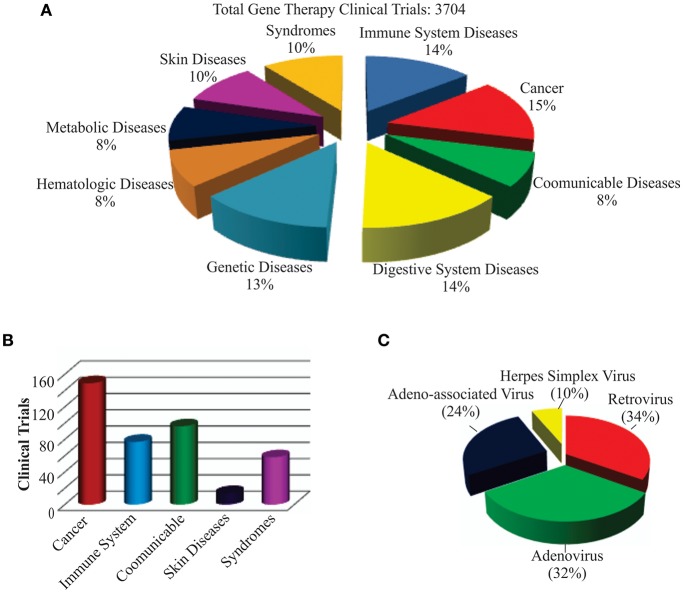
Recent trends in gene therapy research and clinical trials. **(A)** Different diseases being treated by gene therapy in clinical trials. The clinical studies database was searched for the total number of gene therapies conducted in the world to treat different diseases to date. The main focus of the clinical trials was found to be treating cancer, immune, digestive, and genetic diseases. **(B)** Clinical trials actively recruiting patients for testing gene therapy-mediated medicines in curing diseases. This includes both viral and non-viral vector-mediated gene therapies. A relatively large number of clinical trials are recruiting cancer patients for testing different gene therapy-based medicines. **(C)** Different recombinant viral vectors being tested in gene therapy-based treatments.

The world's first gene therapy clinical study was conducted to test a viral-mediated drug at the NIH in 1989, and now 3704 gene therapy studies from 204 countries are listed in the US Government's clinical trials database to date (Figure 4A). More than 50% of them are being conducted in the USA alone. Recently, the US government has removed NIH special oversight rules on gene therapy studies, and the USFDA has decided to consider gene therapy drugs like other medications for approval in order to make gene therapy a therapeutic reality for patients. These clinical studies are testing both viral and non-viral gene therapy drugs to find cures for a wide variety of human diseases, disorders, and infectious diseases. While the majority of these clinical studies are focused on treating cancer, and immune and digestive diseases, skin diseases are yet to receive momentum (Figure 4B). These ongoing gene therapy clinical studies are delivering a wide variety of therapeutic cytotoxic/suicide, tumor suppressor, vaccine antigen, cytokine, receptor, replication inhibitor, and anti-angiogenic genes. Some of the therapeutic genes, vectors, targeted diseases, and their manufacturers are mentioned in Table 1. A large number of non-viral vectors are being used to deliver these therapeutic genes, but viruses dominate as successful vectors in the ongoing clinical studies. The most popular viral vectors being used in clinical studies are AV, AAV, HSV, and RV.

**Table 1:** Naked DNA and viral-mediated drugs in clinical trials.

**Viral Drug/ Intervention**	**Company/Sponsor**	**Gene/Ab/Ligand**	**Disease/Disorder**	**Vector**	**Currentstatus**	**Clinical trial number**
Theragene®	SNUBH	CD/TKrep	Cancer	AV	Phase I	NCT02894944
Ad5-Gag	BDHCMU	Gag	AIDS vaccine	AV	Phase I	NCT02762045
AdMA3	BCCA	MG1MA3	Solid tumor	AV	Phase I	NCT02285816
Ad/L523S	CCF	L523S	Lung Cancer	AV	Phase I	NCT00062907
AdAg85A	MUMC	Ag85A	Tuberculosis	AV	Phase I	NCT02337270
Ad35.CS.01	SUSM	CS.01	Malaria	AV	Phase I	NCT00371189
dAd5GNE	WCMC	GNE	Cocaine	AV	Phase I	NCT02455479
ChAd63-METRAP	CCVTM	METRAP	Malaria	AV	Phase I	NCT03084289
Ad5FGF-4	Angionetics	FGF	Angina	AV	Phase III	NCT02928094
AAV5-hFIX	UniQure	hFIX	Hemophilia B	AAV	Phase I/II	NCT02396342
AAV2-GDNF	NIH	GDNF	Parkinson's	AAV	Phase I	NCT01621581
AAV OPTIRPE65	MEH	OPTIRPE65	Eye Diseases	AAV	Phase I/II	NCT02946879
AAV2hAQP1	NIH	hAQP1	AADC	AAV	Phase I	NCT02852213
rAAV1-PG9DP	SCRC	PG9DP	HIV	AAV	Phase I	NCT01937455
scAVV9.CB.CLN6	NCH	CB.CLN6	Batten Disease	AAV	Phase I/II	NCT02725580
SPK-8011	Spark Thera.	FVII	Hemophilia A	AAV	Phase I/II	NCT03003533
scAAV9.U1ahSGSH	Abeona Thera.	SGSH	MPS III	AAV	Phase I/II	NCT02716246
LentiGlobin BB305	Bluebird Bio	HBB	β Thalassemia	LV	Phase III	NCT03207009
Sin-γ- RV-ADA	BCH	ADA	SCID-X1	γ-RV	Phase I/II	NCT01129544
Anti-MAGE-A3-DP4	NIH	TCR	Cancer	RV	Phase II	NCT02111850
Anti-EGFRvIII CAR PBL	NIH	CAR	Glioma	RV	Phase I/II	NCT01454596
Filgrastim	FHCRC	Filgrastim	FA	RV	Phase I	NCT01331018
MO32(NSC 733972)	UA	IL-12	Gliosarcoma	HSV-1	Phase I	NCT02062827
OrienX010	Oriengene Bio	GM-CSF	Melanoma	HSV-1	Phase I	NCT03048253
HSV1716	NCH	ICP34.5	Neuroblastoma	HSV	Phase I	NCT00931931
NP2	Diamyd Inc.	PENK	Cancer Pain	HSV-1	Phase I	NCT00804076
G207	UA	+ radiation	Brain tumor	HSV-1	Phase I	NCT02457845
SGT-94	SynerGene	RB94	Solid tumors	DNA	Phase I	NCT01517464
CYL2-02	InvivoGen	SST2+DCK	Cancer	DNA	Phase II	NCT02806687

*SNUBH, Seoul National University Bundang Hospital; BDHCMU, Beijing Ditan Hospital of Capital Medical University; BCCA, Vancouver Cancer Centre Vancouver, British Columbia Canada; CCF, Cancer Center of Florida; MUMC, McMaster University Medical Center; SUSM, Stanford University School of Medicine; WCMC, Weill Medical College of Cornell University; CCVTM, Centre for Clinical Vaccinology and Tropical Medicine; MEH, Moorfield's Eye Hospital; AADC, Aromatic L-amino Acid Decarboxylase Deficiency; SCRC, Surrey Clinical Research Centre; NCH, Nationwide Children's Hospital; BCH, Boston Children's Hospital; TCR, T cell receptor; FHCRC, Fred Hutchinson Cancer Research Center; FA, Fanconi Anemia; UA, University of Alabama; Oriengene Bio, Oriengene Biotechnology Ltd; PENK, Preproenkephalin*.

**Table 2:** The cellular and gene therapy products available in the market.

**Drug**	**Company**	**Therapeutic**	**Disease/Disorder**	**Remission**
Luxturna™ Kymriah™ Glybera® Gendicine® Strimvelis™ Oncorine™ Neovasculogen™ SPRS-therapy® laViv™ Provenge™ Imlygic™ Carticel™ Rexin-G™	Spark Therapeutics Novartis uniQure Benda Pharmaceutical GlaxoSmithKline Shanghai Sunway Biotech Human Stem Cell Institute Human Stem Cell Institute Fibrocell Science Valeant Pharmaceuticals Biogen Genzyme Epeius Biotechnologies	*RPE65* CAR-T *LPL* *p53* HSC *p53* *VEGF* Fibroblasts Fibroblasts Dendritic cells ICP34.5 & GM-CSM Chondrocyte Cyclin G1	Inherited blindness Leukemia (ALL) LPLD Head and neck cancer ADA-SCID Head and neck cancer PAD and CLI Skin damage Nasolabial fold Wrinkles Prostate cancer Melanoma Knee cartilage injury Breast cancer, Sarcoma	93% 80% NA 67% 100% NA 90% 75% 57% 38% 50% 92% 40%

*CAR-T, Chimeric antigen receptor T- cell; ALL, Acute lymphoblastic leukemia; LPL, Lipoprotein lipase; LPLD, Lipoprotein lipase deficiency; ADA, SCID—Adenosine deaminase severe combined immunodeficiency; HSC, Hematopoietic stem cell; VEGF, Vascular endothelial growth factor; PAD, Peripheral arterial disease; CLI, Critical limb ischemia; ICP34.5, Infected cell protein 34.5; GM, CSF-Granulocyte-macrophage colony stimulating factor*.

## AV-Mediated Gene Therapies in Clinical Trials

Both AV and RV vectors are being used in more than 50% of the ongoing viral-mediated clinical studies (Figure 4C). The main focus of these are on vaccination and oncolytic therapies. For example, an AV-mediated Theragene (Ad5-yCD/mutTKSR39rep-ADP) delivers a double suicide gene to target stage III pancreatic cancer. AV is being used to deliver the p53 gene in phase II trials to treat recurrent ovarian epithelial, fallopian tube, and primary peritoneal cancer as well as hepatocellular carcinoma (NCT02435186). Also, AV vectors are being used to deliver anti-angiogenic and immunostimulatory genes to treat prostate cancer and malignant pleural mesothelioma (NCT02555397 and NCT01119664). A significant antitumor activity has been demonstrated in phase I-III clinical trials when an AV-based Onyx-015 that undergoes replication selectively in tumors was applied in combination with chemotherapy (14, 191). Vaccination using AV, along with other viruses such as the modified vaccinia Ankara virus (MVA), retrovirus, Sendai virus, and vaccinia virus, is being tested in many clinical trials. AV vectors are also being tested in delivering therapeutic genes for treating malaria, anthrax, HIV, influenza, hepatitis B and C, and severe hemophilia, as well as cardiovascular and many more diseases. AV vectors carrying site-specific endonucleases are being used to edit the CCR5 gene in hematopoietic stem or progenitor cells in AIDS clinical trials (192). The lack of functional dendritic cells in the brain has been attributed to the growth of one of the most aggressive and malignant tumors called gliomas. AV vectors are being used to empower the immune system by expressing the HSV-1 derived thymidine kinase (HSV-1 TK) and cytokine fms-like tyrosine kinase 3 ligand (Flt3L) in the brain. While HSV-1 TK converts ganciclovir into phospho-ganciclovir, a toxic compound to dividing glioma cells, Flt3L differentiates precursors into dendritic cells and acts as a chemokine for dendritic cells resulting into killing of glioma cells and release of tumor antigens in the tumor microenvironment. This follows release of HMGB1, a TLR2 agonist that activates dendritic cells and stimulates dendritic cells loaded with glioma antigens to migrate to the cervical lymph nodes to prime a systemic CD8+ T cytotoxic killing of glioma cells without causing brain toxicity and autoimmunity (193). The median survival of glioma patient is under 2 years and the ongoing clinical trials with DNX-2401, a replication-competent oncolytic AV capable of infecting and killing glioma cells by stimulating an anti-tumor immune response revealed favorable safety profile and prolonged survival of glioma patients (194, 195). Enadenotucirev, a non-natural chimeric oncolytic AV that can retain anti-tumor activity despite intravenous delivery, showed a predictable and manageable safety profile in several advanced cancer patients in phase I clinical studies (196). With encouraging clinical outcome being observed in a large number of ongoing clinical trials, especially in treating cancer, AV-mediated gene therapy is anticipated to make a significant impact on eradicating cancer in the near future.

Although the AV-mediated gene therapy carries a unique advantage over other systems, several concerns must be addressed to offer treatment without side effects. For instance, further improvement in vector development technologies is essential to avoid activation of the endogenous signal transduction pathways and production of cytokines due to anti-vector immune responses that can potentially complicate the clinical outcomes. The necessity of integrin and CAR protein expression on the surface of target cells or tissue to allow efficient infection of AV limits the prospects of treating many diseases. Therefore, generation of novel AV vectors that can infect and transduce target cell or tissue with high specificity, express transgenes up to the therapeutic requirement, induce low organ toxicity and inflammation, and can be detected easily *in vivo* is the need of the hour. Understanding the disease-specific biomarkers, designing and engineering novel AV capsids carrying cell or tissue-specific receptor binding epitopes can reduce the occurrence of unwanted clinical outcome. Since the presence of AV-neutralizing antibodies varies from patient to patient, designing and developing personalized patient-specific capsids can be a promising approach to cure diseases in the future. Development of AV particles that resist inactivation by serum proteins is necessary to promote intravenous administration of therapeutic particles during treatment. Development of strategies to avoid dose-associated toxicity is needed. In addition, contamination with replication-competent virus still remains a serious issue in large scale production of AV preparation for therapeutic purposes (197). Therefore, further advancement in the production of purified AV and AV-based gene delivery technologies is required for using gene therapy to its full potential.

## AAV-Mediated Gene Therapies in Clinical Trials

AAV vectors are being used in more than 200 ongoing clinical studies to treat a wide variety of diseases and disorders worldwide. After the approval of the AAV-based drugs Gendicine and Luxturna, another AAV-based drug is poised to enter the pharmaceutical market in the near future to treat Choroideremia, an X-linked inherited retinal dystrophy that causes night blindness and a constricted visual field. Mutations in *REP1* encoding Rab escort protein 1, a protein involved in lipid modification of Rab proteins, have been implicated in the development of Choroideremia. Patients that received AAV-REP1 therapy showed a significant increase in their visual acuity (198). The product of *CNGB3* provides instructions for making the β-subunit of the cone photoreceptor cyclic nucleotide-gated (CNG) channel, but mutations lead to a defective photoreceptor, decreased visual acuity, and total color blindness, or achromatopsia. In a phase I/II clinical trial sponsored by Applied Genetic Technologies Corporation, AAV was used to deliver *CNGB3* for the successful treatment of achromatopsia (187). AAV is being tested to cure another eye disease, Leber's hereditary optic neuropathy (LHON), a maternally transmitted common mitochondrial disorder caused by point mutations in mitochondrial DNA and impairment of ATP generation. The LHON disease is characterized by apoplectic, bilateral, and severe visual loss. In an ongoing phase I interventional clinical trial, scAAV2 is being used to deliver the P1ND4 gene to rescue visual loss in five legally blind patients (NCT02161380). P1ND4 is a synthetic nuclear encoding gene involved in mitochondrial oxidative phosphorylation. The initial results obtained from this study have showed an improved acuity in two of five patients with no serious adverse events (199). Since treating diseases of the central nervous system is challenging due to the blood brain barrier (BBB), many AAV vectors, especially AAV1, AAV2, AAV5, AAV8, and AAV9, are found to be very useful in transducing neurons (200), and therefore, many AAV-mediated treatments are being tested to cure lysosomal storage disorders, Alzheimer's disease, Parkinson's disease, amyotrophic lateral sclerosis (ALS), epilepsy, spinal muscular atrophy type 1, metachromatic leukodystrophy, aromatic L-amino acid decarboxylase (AADC) deficiency, and Batten disease. Like AV, AAV is yet another useful viral vector for cancer gene therapy. Several AAV vectors are being used to test the expression of anti-angiogenic, cytotoxic, cytokine, and tumor suppressor genes, small RNAs, antigens, and antibodies for cancer cures. A large number of preclinical studies revealed successful treatment with AAV-mediated gene therapy for improved tumor regression (201–206). AAV is considered a powerful vector in targeting the liver for treating hematological diseases. Complete treatment of severe hemophilia B by delivering *FIX* in patients was described as the “holy grail” of gene therapy clinical application (207). In ongoing phase I/II clinical trials, *FVIII* and *FIX* are being delivered to hemophilia A and B patients, respectively (NCT03003533, NCT02484092).

Although AAV vectors are non-pathogenic and safe, and found among the commonly used platforms for gene delivery in preclinical and clinical studies, their potential application in gene therapy is limited by the inability to deliver a therapeutic gene more than 5.0 kb in size, immunogenicity of capsid proteins, difficulty in producing a large supply, requirement of large doses of highly purified vectors, broad tropism, and presence of an extensive anti-AAV immunity in human populations (208–211). Adding empty vectors of AAV to the final vector preparations to serve as a decoy and developing new vectors with high transduction and gene expression potential as well as better understanding of T-cell response to all AAV serotypes in clinical settings would reduce inflammation, immune response, and other viral particle-associated side effects because capsid is the primary interface with the target cell that defines pharmacological, immunological, and molecular properties (150, 207, 212, 213). Therefore, designing and developing more chimeric capsid proteins are critical to generate disease- and cell- or tissue-specific viral particles. For example, substitution of tyrosine to phenylalanine in the AAV capsid protein has enhanced the transduction efficiency with reduced toxicity (214). Better understanding of the underlying mechanisms of intracellular transportation of AAV particles in a disease-specific setting will help developing strategies to improve gene delivery efficacy. AAV vectors are commonly delivered to patients by systemic, intramural, central nervous system, cardiac, and pulmonary delivery but certain sites of the human body elicit no immune response to injection of antigens or viral particles because the BBB prevents the entry of antibodies or resting lymphocytes and the absence of traditional antigen-presenting cells. Therefore, applying AAV particles to patients through immune-privileged sites, such as the central nervous system, mucosal surfaces, eye, placenta, fetus, testicles, and articular cartilage, could be a better option to avoid T-cell response. For example, AAV vectors injected intraparenchymally into the central nervous system to treat Batten's, Canavan, and Parkinson's diseases showed little or no adaptive immune response in many clinical trials (155, 215–218). Monitoring T-cell response in patients by using advanced tools especially multicolor flow cytometry, mass cytometry, and enzyme-linked immunospot (ELISpot) assay will minimize the risk of the unexpected clinical outcome (209). Also, for reduced T-cell response and optimal expression of a therapeutic gene, intramuscular instead of systematic injection of AAV particles is recommended because healthy muscles express only low levels of MHC class I antigens (209, 219). Use of immunosuppressive drugs was found safe to maintain therapeutic gene expression in many clinical trials (150, 220, 221), and their use could be an option for better clinical outcome, but maintenance of transgene expression remains unpredictable. Although AAV offers the expression of a therapeutic gene for nearly 1 year without integrating into the host's chromosomes, applying CRISPR/Cas9 technology would resolve long-term expression and mutagenesis issues. Production of high titers of purified AAV particles by employing ionic iodixanol gradients and ion exchange chromatography instead of using the toxic CsCl is also important for the success of gene therapy (222, 223). Recent developments in the production of high quality AAV particles from transfection efficient HEK293 cell suspensions in shaker flasks and WAVE bioreactors free of all animal and human products will certainly improve the success of gene therapy application (224). This system was further improved by changing the NaCl concentration in the medium and optimizing conditions for Expi293F cell infection by helper herpes simplex virus (HSV) (225). However, contamination of the final AAV particle preparation with HSV cannot be ruled out. The AAV particles generated from the baculovirus expression system carry low levels of VP1 capsid protein, so high doses are used in clinical trials to increase transduction efficiency at the expense of immune response (226). No disease caused by AAV infection has been reported to date but repression of PPP1R12C gene promoter in host cells by the rep proteins of AAV2 is clearly a concern (227). Therefore, more efforts are necessary to smooth out the landscape surrounding AAV for its more pronounced clinical benefits in gene therapy.

## HSV-Mediated Gene Therapies in Clinical Trials

More than 90 gene therapy clinical trials have been conducted using HSV as a vector to deliver therapeutic genes for curing various diseases to date. They have been extensively used for tumor therapy and vaccine development. After the advent of HSV-based T-VEC drug for melanoma treatment, many HSV vectors are being used to deliver suicide genes to treat anaplastic thyroid cancer (228). Since immunotherapy is currently a hot topic in cancer research and gaining more attention; oncolytic viruses are often combined with immune checkpoint blockades such as T-VEC combined with anti-PD1 Pembrolizumab, anti-CTLA-4 Ipilimumab, and neoadjuvant to increase their therapeutic potential (68). Also, the oncolytic HSV-1 carrying four copies of miR-145 target sites combined with radiation has been shown to be more effective than radiation alone (122, 229). A current phase I clinical trial uses an engineered HSV rRp450 designed to kill cancer cells in order to treat liver metastases and primary liver tumors (NCT01071941). HSV is also used as a transneuronal tracer defining connections among neurons by virtue of traversing synapses (230). HSV has much potential in treating problems of the urinary system. A recent study reports HSV-1 as a vector for delivering poreless TRPV1 channels or protein phosphatase 1α to reduce bladder over-activity in rats (231). HSV-mediated treatment also alleviated bladder pain. These have the potential to offer treatment to cases of overactive bladder (OAB) and interstitial cystitis/bladder pain syndrome (IC/BPS). However, infectivity of solid tumors, leakage, off target viral replication, sequestration, and delivery methods are still hampering the progress of HSV-mediated oncolytic viral therapy. Although the nervous system is the natural site for HSV latency, the full potential of HSV-mediated gene therapy in treating nerve diseases is yet to be discovered. Several studies treating chronic pain were successful in animal models but very few have reached clinical trials to date. HSV vectors have certainly promising perspectives in clinic trials but detailed understanding of virus-host interaction will minimize cytotoxicity and biohazards generation. Recently, strategies to develop transduction efficient, alternate vector entry and transcriptionally retargeted oncolytic HSV viruses were reviewed (232–234). The therapeutic potential of amplicons is still undermined by production and stability issues; therefore, focus needs to be on improving vector design, construction, and production technology. Developing new HSV vectors carrying genes that enhance tumor cell lysis will increase oncolytic therapeutic efficacy. While gliomas do not express miR-124, it is highly expressed in normal brain, and designing HSV vectors carrying the same could be a promising approach to treat glioma. The full potential of expression libraries created by using HSV vectors in regenerative medicine is yet to be seen in curing human diseases (235). Since oncolytic virus therapy is considered a major breakthrough in treating cancer after the success of radiation and immunotherapies, development of safe and tumor-selective new HSV vectors is necessary for its promising future. Optimizing vector delivery methods especially to solid tumors and in immune-compromised patients will certainly improve oncolytic viral therapy. Exploring their roles in gene editing and repair will expand the horizons of gene therapy.

## RV-Mediated Gene Therapies in Clinical Trials

RV vectors can be applied to cure a wide variety of diseases and disorders such as cancer, HIV, ADA-SCID, melanoma, WAS, and many others. Though the majority of retinal gene therapy trials use AAV, some use lentivirus because of its larger gene capacity. For example, Usher syndrome causes hearing loss, less vestibular function, and a pigmented retina (187). Currently, a phase II trial is underway to use lentivirus to deliver a 5.0-kb *MYO7A*. Additionally, a phase II trial that is projected to deliver *ABCA4* by lentivirus to treat Stargadt disease, an inherited macular degeneration that causes cell degeneration, is underway (187). Furthermore, lentivirus is a favorable vector to treat sickle cell anemia because of the advantages it offers, including a large transgene capacity, stable long-term expression, and safer integration (236). A single base substitution in the β-globin gene causes the erythrocyte sickling characteristic of sickle cell anemia. Treatments for sickle cell anemia are transitioning into self-inactivating lentivirus with a deletion in the U3 region of the 3' LTR, which has a safer integration profile (236). A clinical trial sponsored by Bluebird Bio used LentiGlobin BB305, which delivered β-globin T87Q. Clinical results showed 24% anti-sickling (NCT03207009). For treating immunodeficiency, there have been adverse effects reported in the past by gammaretroviral vectors. In the treatment of X-linked SCID, CD34+ hematopoietic stem cells were transduced with murine gammaretroviral vector, which led to an increase in immune function, but 5 patients developed T cell leukemia because of insertional mutagenesis into oncogenes (185). In the treatment of WAS, a gammaretroviral vector expressing *WAS* transgene delivered to patients caused 7 out of 10 to develop leukemia (185). Recently, self-inactivating lentivirus was used to treat five patients with X-linked SCID. Two patients had restoration of immune function even 2–3 years after treatment (237). A current phase I/II clinical trial is using a self-inactivating gammaretrovirus to treat SCID-X1 (NCT01129544). Other current clinical trials include a phase II trial using a retroviral vector to transfer ADA into hematopoietic stem cells to treat ADA-SCID (NCT00598481). A replicating Toca 511 RV vector is being used in a phase I trial to treat recurrent high-grade glioma (NCT02598011). RV is being used in a phase I/II trial to transduce white blood cells with the CAR-T cell receptor to target mesothelin for patients with metastatic cancer (NCT01583686). Donor T cells are being transduced with RV to express the caspase-9 suicide gene in a phase I trial to treat cancer (NCT01494103). Duchenne muscular dystrophy occurs when a lack of dystrophin expression causes muscle degeneration. In a proof-of-concept study, the full-length sequence of dystrophin was spread over two co-packaged RNA copies and delivered via a lentiviral vector. The vector integrated and gave long-term expression of dystrophin (238). Additionally, a RV vector expressing MazF endoribonuclease is being used to transduce CD4+ T cells to treat HIV in a phase I trial (NCT01787994). AIDS-related non-Hodgkin lymphoma is being treated in a phase I clinical trial that transduces stem cells with genes for HIV RNA using lentivirus in order to evoke an immune response (NCT01961063).

Since immunity is the primary barrier for the success of viral gene therapy, it is critical to design viral vectors that can subvert the complement system. The LTRs of RV serve as promoters, enhancers, binding sites for various nuclear proteins, chromatin modulators, and polyadenylation signals. Therefore, applying genetic engineering and CRISPR technology will avoid exacerbating the insertional mutagenesis issue. This issue can also be avoided by using non-integrating RV vectors or integrase inhibitors during treatment. The RV-mediated gene therapy will immensely benefit from developing technologies to guide and monitor transgene insertion in the host cell chromatin. Although RV vectors can deliver a transgene up to 10 kb in size, production of high titer virus, chromatin structure, and epigenetic modification near the insertion site still remain issues in clinical applications. Thus, better RV vectors are needed for future gene therapy applications. Since viral infection depends on the expression of target cell surface receptors and viral envelope protein, designing and constructing to produce efficient and cell-, tissue- and disease-specific recombinant RV vectors are necessary to obtain the expected clinical outcome. New RV vectors with optimized LTRs, created by replacing promoter/enhancer elements with cell- and tissue-specific promoters and enhancer sequences, will boost their use in curing many diseases with fewer or no side effects. Novel RV vectors are needed to transduce heart and other body organs for their wide spread use in gene therapy. Introduction of miRNA binding sites in the viral RNA has been suggested to control posttranscriptional regulation of disease-causing genes (239). The use of advanced RV vectors carrying the woodchuck posttranscriptional regulatory element (wPRE) to increase transgene mRNA stability, export, and translatability will help to accomplish better clinical outcome (240). As delivering genetic information in the form of RNA is an increasingly popular method, RVs carrying no RT or integrase are poised to play a significant role in a gene editing, vaccination, tumor therapy, gene therapy, transdifferentiation, reprogramming, and other biotechnological applications in the near future (241).

## Risks Associated With Viral Vectors

Since an estimated 10^31^ virus-like particles exist on the Earth and they are present in the blood, nose, mouth, lung, vagina, gastrointestinal tract, conjunctiva, skin, and the mammalian genome, viruses appear to play a major role in human life (242, 243). The general concerns with viral vectors are the risks of an immune response, off-target effects, inflammation, and insertional mutagenesis. An immune response could make a viral treatment less efficient, or the resulting creation of antibodies could preclude a second dosage of the same virus (244–248). Inflammation was seen as a worst-case scenario in the 1999 death of Jesse Gelsinger caused by a very high dosage of adenovirus (249). Tailoring the viral dose to the patient, however, can better control this risk. Also, insertional mutagenesis is a major obstacle that the gene therapy field must overcome. The risk of inserting a gene into a tumor suppressor gene or activating an oncogene is present for the vectors that integrate into the unwanted locations of the genome, such as retrovirus. To counter this, vectors can be used that do not integrate readily into the genome. Additionally, self-inactivating vectors can be manufactured that do not contain their own promoter; rather, another internal promoter in the cell is used. This leads to less genotoxicity and is a safer alternative to traditional integrating vectors (52). Other concerns are that viral vectors are only relevant for monogenic disorders because of their limited DNA-carrying capability. However, HSV-1 is an example of a virus that has enough carrying capacity for multiple genes. Additionally, dual vector systems, such as dual vector adeno-associated virus, have larger transgene capacities. Also, finding the appropriate virus to infect the desired cells is often difficult, and there is the risk that the virus could cross the Weismann barrier and infect germ line cells. Furthermore, viruses are generally susceptible to genetic variations. Integration into undesirable sites such as regulatory, oncogenes or tumor-suppressor genes would be undesirable. Deletion of virulence genes may affect their ability to infect or integrate with the host chromosome, thus compromising their effectiveness as vectors. Additionally, a social stigma is associated with viral therapy. Most patients would be concerned about being infected by a live virus—a concern also held about viral vaccines. Since their ubiquitous presence is a reality, why shouldn't humankind start accepting them as wonderful molecular biological tools with which to build novel and powerful medicine?

## Challenges and the Way Forward

Since its birth in the 1960s, gene therapy has come quite a long way by providing an alternate one-time treatment option for cancer, metabolic disorders, and neuronal, immune, and infectious diseases. Notably, it has been able to treat beta thalassemia, Leber's congenital amaurosis, severe immunodeficiency diseases such as ADA-SCID, and more. However, the full potential of gene therapy is yet to be witnessed in regenerative medicine, a branch of translational medicine where engineering or regenerating human cells, tissue or organs enables restoration or establishment of normal function. With recent impressive results observed in vaginal gene therapy in preclinical trials, gene therapy is poised to enter the clinical phase for treating infectious diseases in the near future (250). Both viral and non-viral vectors can be used to deliver DNA, each of which has its own advantages and disadvantages. Additionally, genome-editing technology is an up-and-coming method of delivering DNA to specific parts of the genome. With all of these breakthroughs have come hurdles, such as the death of Jesse Gelsinger in 1999 and the development of leukemia in patients who have been treated for WAS and ADA-SCID. The ethical concerns of patients must be heeded as well. However, these challenges do not reflect a flaw in the concept. Simply, more research is needed to avoid technical issues such as the production of viral particles in large scale, formulations for long-term storage stability, immune responses, and insertional mutagenesis. Loading of viral particles with a therapeutic gene during production is mostly done by transient transfections, a rate-limiting step in large scale production of viral particles. Alternate approaches such as stable cell lines expressing capsid proteins and insect cells based baculovirus expression systems would be useful for mass production of viral particles. This underdeveloped modern medicine needs discovery and engineering of better viral vectors to deliver therapeutic genes precisely to the target diseased cells or tissue.

Gene therapy is a rapidly expanding field, and it seems that scientists have only scratched the surface of its potential. The more that is discovered about how to optimize gene delivery vectors, the closer this field gets to delivering wide-scale solutions to modern medicine. The future of gene therapy moves toward engineering safer and more efficient vectors, combining multiple existing strategies such as viral vectors with genetic engineering technologies, and personalizing all characteristics of gene therapy treatments to the patient, as it has been shown that host genetic variants affect the efficacy of vector-mediated gene delivery (251). This includes understanding of the repertoire of receptors on a target cell in diseased conditions to help in designing appropriate capsid proteins for viral particles. Although the full panoply of gene therapy's might is yet be witnessed, it has enormous potential to shed light on human afflictions, add value to patients' lives, and contribute to future economic growth. Although gene therapy currently shares less than one percent of the total $1.2 trillion world annual pharmaceutical market, it is expected to create approximately a $12 billion market in the next 10 years. According to a market research and advisory company, Allied Market Research, cancer gene therapy alone has created a $289 million market in 2016 but it is expected to reach $2,082 million by 2023. Gaining popularity among the global medicinal community, gene therapy has become an attractive market for companies and investors. However, the ethical acceptance and advancement in the technology to avoid unwanted clinical outcomes are critical for driving its market growth. Also, the translation of laboratory studies to animal studies and then to clinical trials is a long, tedious, and expensive process to ensure the safety of patients. As a result, if the USFDA, with its patchy regulations, continues its approval rate, providing gene therapies for all the genetic diseases will take many years to come. Therefore, a new perspective on creating a conducive atmosphere for improving this modern cutting-edge gene therapy technology is necessary to transform the lives of patients with severe genetic illnesses, infectious diseases, and disorders. As mentioned elsewhere, knowledge has no boundaries, and there exist unlimited methods to develop a novel invention; every bump in the investigating path can be considered an inspiration and source of energy to advance research, a never-ending learning process.

## Author Contributions

VB and RG conceived the concept and wrote the manuscript, and the other authors listed made substantial, direct intellectual contributions to the work, and approved it for publication.

### Conflict of Interest Statement

The authors declare that that they have no affiliations with or involvement in any organization or entity with any financial interest (such as honoraria; educational grants; participation in speakers' bureaus; membership, employment, consultancies, stock ownership, or other equity interest; and expert testimony or patent-licensing arrangements), or non-financial interest (such as personal or professional relationships, affiliations, knowledge or beliefs) in the subject matter or materials discussed in this manuscript.
